# Sequencing, Assembling, and Correcting Draft Genomes Using Recombinant Populations

**DOI:** 10.1534/g3.114.010264

**Published:** 2014-02-13

**Authors:** Matthew W. Hahn, Simo V. Zhang, Leonie C. Moyle

**Affiliations:** * Department of Biology, Indiana University, Bloomington, Indiana 47405; † School of Informatics and Computing, Indiana University, Bloomington, Indiana 47405

**Keywords:** genome, genetics, assembly, next-generation sequencing, duplication

## Abstract

Current *de novo* whole-genome sequencing approaches often are inadequate for organisms lacking substantial preexisting genetic data. Problems with these methods are manifest as: large numbers of scaffolds that are not ordered within chromosomes or assigned to individual chromosomes, misassembly of allelic sequences as separate loci when the individual(s) being sequenced are heterozygous, and the collapse of recently duplicated sequences into a single locus, regardless of levels of heterozygosity. Here we propose a new approach for producing *de novo* whole-genome sequences—which we call recombinant population genome construction—that solves many of the problems encountered in standard genome assembly and that can be applied in model and nonmodel organisms. Our approach takes advantage of next-generation sequencing technologies to simultaneously barcode and sequence a large number of individuals from a recombinant population. The sequences of all recombinants can be combined to create an initial *de novo* assembly, followed by the use of individual recombinant genotypes to correct assembly splitting/collapsing and to order and orient scaffolds within linkage groups. Recombinant population genome construction can rapidly accelerate the transformation of nonmodel species into genome-enabled systems by simultaneously producing a high-quality genome assembly and providing genomic tools (*e.g.*, high-confidence single-nucleotide polymorphisms) for immediate applications. In populations segregating for important functional traits, this approach also enables simultaneous mapping of quantitative trait loci. We demonstrate our method using simulated Illumina data from a recombinant population of *Caenorhabditis elegans* and show that the method can produce a high-fidelity, high-quality genome assembly for both parents of the cross.

Whole-genome sequence information has transformed research in traditional model systems, and now next-generation sequencing (NGS) technology is at a turning point in its application to nonmodel organisms (Tautz *et al.* 2011). Researchers working in data-rich, but sequence-poor, organismal systems are eager to apply these technologies to their own species. However, the challenges involved in generating useful, high-quality, whole-genome sequences in these naïve systems are substantial. Nonmodel systems can have biologic features—enforced outbreeding, absent or sparse linkage maps, complex genome structures, large numbers of repetitive elements, etc.—that directly hamper *de novo* assembly and therefore the general availability of genomic tools.

Of the challenges inherent to generating a *de novo* genome sequence from nonmodel species, the generation time and the reproductive biology of many of these species impose especially important constraints on making accurate assemblies. The inability to make highly inbred individuals and/or to use a single individual as a reference genome frequently means that there will be a large amount of variation among the assembled alleles (*i.e.*, haplotypes). This variation (present as heterozygosity in a single individual) is challenging for standard assembly approaches because it is difficult to distinguish allelic sequences from truly paralogous loci that have low divergence between them. One consequence of these errors is large-scale, systematic bias in assembly and annotation. The problem is especially severe for NGS technologies because of the short nature of the reads (Alkan *et al.* 2011; Schatz *et al.* 2010). The result of such “allelic splitting” is to increase the total size of initial genome assemblies (*e.g.*, Barrière *et al.* 2009; Cheung *et al.* 2003; Holt *et al.* 2002). Even in genomes assembled from highly inbred, but not completely homozygous, individuals, large numbers of split alleles will falsely inflate the apparent number of new gene duplicates (*e.g.*, Colbourne *et al.* 2011; Denoeud *et al.* 2010). One approach to dealing with the problem of allelic variation is to allow greater numbers of mismatches between the sequences combined together into a single locus during the assembly process (*e.g.*, Dehal *et al.* 2002). Unfortunately, the effect of this approach is to make it more likely that true paralogs will be collapsed into a single locus, causing an underestimation of the number of recent duplication events and erroneously decreasing the apparent size of the genome. The collapse of highly similar paralogous loci can occur during assembly regardless of levels of heterozygosity (*e.g.*, Lander *et al.* 2001) but is an especially acute problem when assemblies must also account for high levels of allelic variation. Therefore, an overall challenge of any *de novo* genome sequencing project is to find the right balance between the splitting and collapsing of loci and to apply appropriate *post hoc* methods that can be used to identify such errors (Bailey *et al.* 2002; Kelley and Salzberg 2010). This challenge is likely to be particularly acute in emerging nonmodel systems.

The availability of completely inbred (or haploid) individuals does not solve all of the problems of either genome assembly or genome-enabled science. One irony of genomics in model organisms is that although the use of nearly homozygous strains has allowed researchers to generate highly accurate reference genomes, these sequences contained no data on within-species variation (*e.g.*, Adams *et al.* 2000; *C. elegans* Sequencing Consortium 1998). Because variation data—often in the form of single nucleotide polymorphisms (SNPs)—are necessary for genetic mapping, studies of population structure, and many other purposes, this has left these systems at a disadvantage relative to nonmodel organisms in which SNPs were identified during the genome sequencing process. Obtaining SNPs in model organisms has required further sequencing efforts (*e.g.*, Wicks *et al.* 2001), some of which did not come about for nearly a decade after the initial genome release (Langley *et al.* 2012; Mackay *et al.* 2012; Sackton *et al.* 2009). Moreover, even when SNPs are present in the individual sequenced to make the reference genome, the haplotypic phase among alleles is still not usually known, except in exceptional circumstances when multiple SNPs are contained within single sequence reads or paired reads (Bansal *et al.* 2008; Kim *et al.* 2007; Kitzman *et al.* 2011). That is, the arrangement of alleles on an individual’s maternal and paternal chromosomes is not known in most current genome assemblies.

Finally, even when a reasonable assembly of sequence reads can be performed, standard genome sequencing is unable to place scaffolds onto linkage groups without substantial additional investment in approaches such as fluorescent *in situ* hybridization, genetic mapping (*e.g.*, Hyten *et al.* 2010), optical mapping (*e.g.*, Zhou *et al.* 2009), or, when possible, radiation hybrid mapping (*e.g.*, Lander *et al.* 2001). Without the ability to place sequences on a physical map, genome sequences contain little information about synteny, the colocalization of genes with quantitative trait loci (QTL), the content of any sex chromosomes, or the presence of inversions (see Lewin *et al.* 2009 for additional shortcomings). In addition, the distance between duplicated sequences cannot be estimated, and therefore no inferences about duplication mechanisms or the effects of physical distance on either the probability of interlocus gene conversion (*e.g.*, Casola *et al.* 2010) or adaptive evolution (Han *et al.* 2009) can be made.

Here we propose a new method for producing whole-genome sequences that solves many of these problems. Our method involves sequencing a population of recombinant individuals from a known crossing design, and we therefore refer to it as recombinant population genome construction (RPGC). The basic idea of RPGC is to barcode and sequence (using the most up-to-date NGS technologies) a recombinant set of individuals from a known crossing design to generate the reads used for genome assembly. This set of total reads can be used together for the initial *de novo* genome assembly, but each individual’s genotype also can be determined by examining reads with the appropriate barcode. The availability of individual recombinant genotypes makes it possible to address many problems in genome construction, including the assignment and ordering of scaffolds to linkage groups, the ability to distinguish alleles from paralogs, and the identification of a high-quality set of markers. In addition, our approach allows researchers to infer the phase of whole chromosomes in the parents of the cross and to map the location of loci underlying any phenotypes segregating in the cross. Herein we describe the method in greater detail and present the analysis of an idealized simulated dataset that demonstrates the power of the method. As we discuss, because RPGC is flexible in its application to a wide range of organismal systems, its primary anticipated application is to whole-genome sequencing and assembly in traditionally nonmodel organisms.

## Materials and Methods

**Figure 1:**
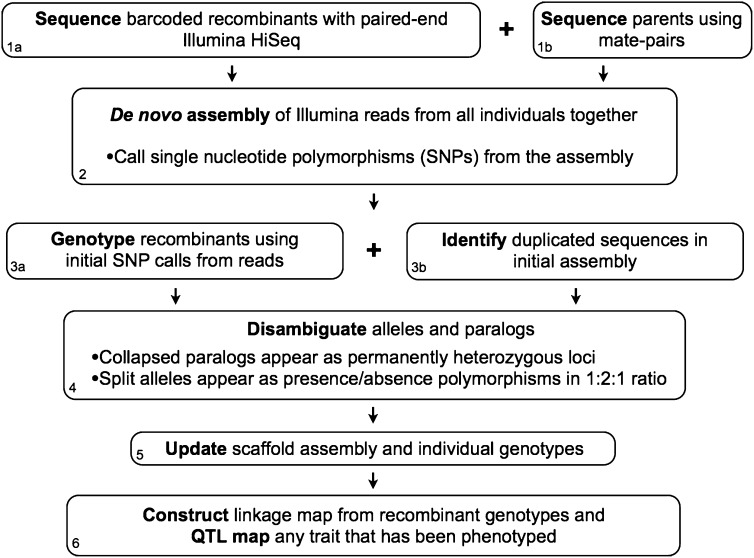
General outline of the steps involved in recombinant population genome construction. Each individually numbered step is described in detail in the text.

Figure 1 provides an overview of the steps involved in RPGC. By iteratively assembling reads, calling genotypes, and resolving assembly inconsistencies (*i.e.*, alleles that have been split into multiple loci, or multiple loci that have been collapsed into one), RPGC combines multiple genome construction steps—each of which has been achieved on its own—into one sequencing approach. This integrated approach not only saves money and time but also promises to provide a much higher quality finished product. Here we outline the proposed steps using the case of two inbred parental strains that are crossed together, with the F_1_ self-fertilized to generate F_2_s, although this approach can be used in a much wider range of offspring population structures and therefore biological situations (further addressed in the *Discussion*). Throughout, we identify instances in which crossing-design considerations will influence the difficulty of individual steps. Although the method we describe is not tied to any specific NGS technology, we will generally refer to the constraints of the Illumina HiSeq platform because of its relative ubiquity and low cost. In the *Results*, we carry out the steps described here on a simulated dataset by using a recombinant inbred line (RIL) mapping population.

### Step 1

The method starts with the sequencing of the F_2_ population (1a) and the parents of the cross (1b). As with all next-generation genome assembly projects, at least two classes of sequences, with different insert lengths, should be used to generate long scaffolds (Gnerre *et al.* 2011). This combined approach ensures adequate depth of sequence coverage (using cheaper, short-insert technologies) to make contigs, in addition to smaller numbers of long reads (using more expensive long-insert or long-read technologies) to enable assembly of these contigs into scaffolds. For RPGC, given the relatively cheaper library costs for short-insert (paired-end) Illumina sequencing, it is most cost-effective to sequence the recombinant population (step 1a) using this (or equivalent) short-read technology. With the current sequencing power of HiSeq, a large number of recombinants can be barcoded and sequenced within a single flowcell lane, and a few lanes in total might be sufficient for a genome project. The number of F_2_s that must be sequenced will differ depending on the crossing design and a number of other factors (as discussed herein in *Methodological trade-offs*). In contrast, because of the greater costs of individual long-insert libraries and long-insert (*e.g.*, Illumina mate-pair) or long-read (*e.g.*, PacBio) sequencing technologies, these methods should likely only be used to sequence the parents of the crosses (step 1b) to increase scaffold lengths. These recommendations are obviously subject to modification with evolving technologies and costs. In some outbred experimental designs, it also may be advisable to sequence F_1_s rather than (or in addition to) parents in step 1b, because only a subset of parental alleles will be present in the F_2_ generation.

### Step 2

Using data from steps 1a and 1b, we can generate an initial genome assembly and identify SNPs. The basic steps of the initial genome assembly under RPGC mirror those taken for a standard sequencing project. The most up-to-date assembly programs (*e.g.*, ALLPATHS-LG; Gnerre *et al.* 2011) can be used to jointly assemble the paired-end and long-insert/long-read sequences. One important aspect of assembly quality is total sequencing coverage. Total coverage in RPGC is equal to (# F_2_s)*(coverage/F_2_), so that 5× coverage for each of 96 F_2_s would give 480× total coverage for the genome assembly. Adding in the long-insert/long-read sequencing of the parents increases overall coverage and ensures long contigs and scaffolds. One consideration with standard next-gen assembly software is that too much coverage can actually harm assembly quality, that is, extremely high coverage can introduce a non-negligible number of errors into assemblies and assembly quality can suffer, or at least not continue to improve (Haridas *et al.* 2011; Lin *et al.* 2011). For this reason, *de novo* assembly might use all of the long-insert/long-read data plus only a subsample of the paired-end reads from F_2_s. The quality of the initial genome assembly will depend not only on the number and length of reads but also on the number of variable sites between the parents (higher variant density likely means worse initial assembly). Although this finding suggests that the optimal choice for this step is to minimize variation in the sequenced population, downstream assembly, correction, and mapping steps actually benefit from intermediate levels of variation between parents of the recombinant population (as we discuss herein).

The second major analysis to be conducted in step 2 is to identify SNPs (or any other variation—we will refer only to SNPs for simplicity) from the total pool of sequences used for assembly. In an F_2_ population constructed from inbred parents, we expect informative variable sites to be at frequency 50%. For other crossing designs, and for sex-specific and sex-linked regions, expected allele frequencies come from equivalent Mendelian predictions. In crosses involving species with sex chromosomes, ensuring that half the recombinants come from each sex will make it easier to identify sex-linked scaffolds. In all crosses, the deep sequencing coverage of the initial assembly can be used to identify sites that have only two alleles present, with each allele represented by multiple reads with high-quality scores. One can also restrict analyses to variable sites with both alleles represented by 50% of the reads (± allowable error), to ensure informative markers.

### Step 3

Using the SNPs identified in earlier steps, we can begin to assign genotypes and to identify/differentiate allelic from paralogous variation. After finding variable positions, each individual F_2_ must be assigned a genotype (homozygote for either allele or heterozygote) at each site (step 3a). The accurate assignment of genotypes for each variable site will largely be determined by the read coverage at that site within each recombinant. This step can also take advantage of software that takes into account the prior knowledge that a site is variable when calling genotypes (*e.g.*, GATK; McKenna *et al.* 2010); this ensures more accurate genotype calls. Either in this step or in later, updated, genotyping steps, multiple variable sites within a single scaffold can be combined to infer more accurate genotypes.

Simultaneously, RPGC also aims to identify all possible allele-splitting events and paralog-collapsing events for later testing (step 3b). To find the possible split alleles, one must first find all highly similar paralogous loci in the initial assembly. The most straightforward way to do this is the whole-genome assembly comparison (WGAC) approach (Bailey *et al.* 2001). This method identifies all pairs or larger sets of windows with high sequence similarity (>90%); these are set aside for downstream testing to evaluate whether they are truly split alleles. One can also use the read-depth at loci throughout the genome to help identify split alleles and collapsed paralogs, an approach taken by the widely used whole-genome shotgun sequence detection (WSSD) method (Bailey *et al.* 2002). After mapping all reads against the initial assembly, split alleles should show 50% of the average read-depth if a single locus is split into two loci; percentages can be lower than 50% if more than two alleles are present in the cross and are therefore split into more than two loci. Conversely, two collapsed paralogs should show 200% of the average read-depth, because all of the reads from two physical loci will be mapped to one locus in the assembly. More than two paralogs can also be collapsed, with commensurately higher read-depth.

### Step 4

**Figure 2:**
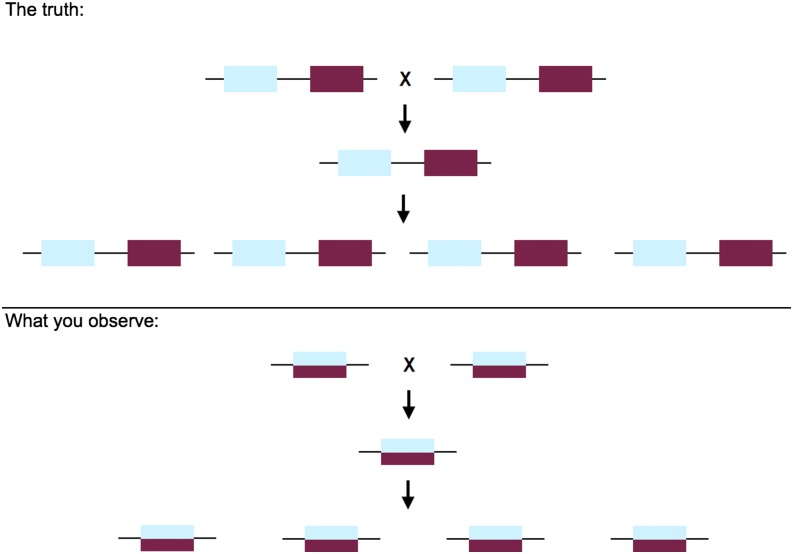
The pattern of segregation of collapsed duplicates through an F_2_ cross. The top panel shows the physical reality of genes arranged on chromosomes, with two duplicates present in each parent, F_1_, and F_2_. The bottom panel shows how, when duplicates are collapsed into a single locus, all individuals appear to be heterozygous at sites that differentiate the two copies.

**Figure 3:**
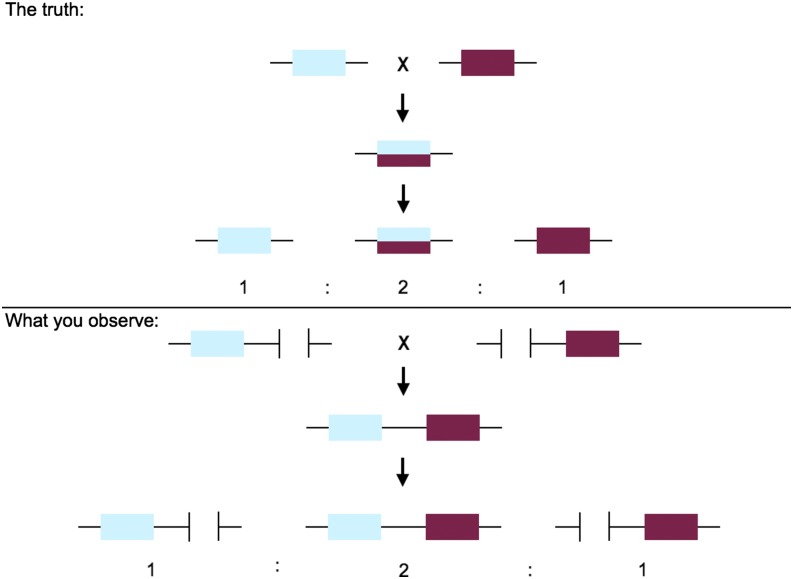
The pattern of segregation of split alleles through an F_2_ cross. The top panel shows the physical reality of a single gene that differs in allelic sequence between the parents. The F_1_ and half the F_2_s are heterozygous. The bottom panel shows how, when alleles are split into two loci, each parent appears to be missing a locus, whereas the F_1_ and half the F_2_s have both loci present.

Using information about the segregation of variants in the recombinant population, we can then use RPGC to refine the identification of alleles *vs.* paralogs. Given an initial *de novo* assembly and initial genotypes for each recombinant individual at all variable sites, we can determine whether variants segregate as a single locus or multiple loci. This step allows us to get “true” loci that can be used to both improve the assembly (step 5) and to construct a linkage map (step 6). To distinguish alleles from paralogs, one can use genotypic proportions among the F_2_s to detect deviations from expected values. For example, for inbred parents there are straightforward genotypic expectations for both split and collapsed loci. For two or more duplicates that have been collapsed (assuming there is a fixed difference between them), all of the F_2_s will appear to be heterozygous at positions that distinguish the paralogous loci (Figure 2). This pattern is not expected—and can easily be rejected statistically—with even a modest number of F_2_s. Conversely, for one locus with alleles that have been split into two loci, we expect each of the two loci to segregate as presence/absence in a 1:2:1 ratio (Figure 3). This pattern will never be seen for two loci that exist in a physical genome, even if there are copy-number variants segregating in the cross. The “presence” and “absence” of these loci will only manifest itself at sites that differ between true alleles (*i.e.*, sites that are truly heterozygous), so these distinguishing sites must be determined beforehand in order to quantify segregation patterns. For both collapsed and split loci we expect read-depth to be high or low, respectively, as described in the previous step; this information can be used to bolster confidence in the inference of split or collapsed loci.

### Step 5

Once split alleles and collapsed paralogs have been identified, one can use these data to iteratively improve the assembly and update genotype calls at each locus. Assembly software makes several typical decisions regarding the placement of split alleles—it generally places them in tandem to one another (*e.g.*, Holt *et al.* 2002) or it places one on a miniscaffold by itself (*e.g.*, Colbourne *et al.* 2011). In both of these cases either the two tandem loci can be collapsed into one in the updated assembly, or the copy on a mini-scaffold can be removed from the assembly altogether. If the two loci have been assembled onto equally supported larger scaffolds, one can use the genotype information contained within both loci together to determine the true location during the linkage-mapping step. That is, given the pattern of segregation shown in Figure 3, one can infer the true genotypes for each individual recombinant at the single-locus that has been split, and these genotypes can be used to place the locus in the larger assembly. For separate loci collapsed in the initial assembly, further local assembly may be needed to find paired-end information that reveals the actual location of the collapsed locus or loci. These correction steps should be carried out on all the problematic loci identified in step 4. Given an updated assembly the genotypes at all variable sites can also be updated, in all recombinants and parents.

### Step 6

With an updated assembly and updated genotypes, scaffolds can be placed onto a linkage group—and ordered and oriented within linkage groups—with the use of standard map construction methods. Only scaffolds with variable sites can be mapped, but increased scaffold lengths due to high coverage and long-insert/long-read sequencing will ensure that most will contain at least one such site. As an additional potential bonus of the linkage mapping step, it should be possible to reconstruct whole parental haplotypes. For crosses between two inbred strains, this means that one should be able to reconstruct two entirely separate genomes—one for each parent. Finally, with the fully genotyped set of recombinant individuals and parents, one can easily QTL map any phenotypes that have been scored.

## Results

To demonstrate the power of RPGC, we simulated Illumina sequence reads from an RIL crossing design using the *Caenorhabditis elegans* genome as a template. This simulation represents a highly idealized dataset because RILs simplify genotyping expectations (they should always be homozygous at every locus) and because the *C. elegans* genome is quite small (~100 Mb) and individuals can self-fertilize. Nevertheless, this dataset allows us to demonstrate the methods outlined previously while presenting a best-case scenario for the success of RPGC. Throughout the pipeline described herein, we have had to make decisions about acceptable minimum quality scores for calling variants, as well as many other important assembly and mapping parameters. Although these do not necessarily represent the optimal parameter choices for each particular step, they are typical choices used by researchers in the field. Similarly, we have taken advantage of software that is commonly used in the field, although RPGC is not tied to specific software packages or algorithms. A manual detailing the software and specific commands used at each step is available in the supporting materials (Supporting Information, File S1); all of the scripts written specifically for RPGC are also available from our website at http://sites.bio.indiana.edu/~hahnlab/RPGC.html.

### Implementing RPGC on a simulated recombinant population

We used the reference *C. elegans* strain N2 as one parental genome and simulated a second parent that had 120,618 single nucleotide differences and 20,273 short indel differences from N2 spread across the genome randomly; we refer to the second parent as N2b. This level of diversity approximates that seen between two divergent strains of *C. elegans* (Wicks *et al.* 2001). We simulated individual RILs equivalent to eight generations of selfing by a set of individual F_2_s that were produced from crossing perfectly isogenic parental strains and self-fertilizing the resulting F_1_. We assumed that this resulted in a total of five crossing-over events per chromosome in the sequenced RILs and that recombination events were uniformly distributed along chromosomes. For 70 female RILs and the two parental strains, we simulated 5× sequence coverage of 2×100-bp paired-end Illumina reads from 180-bp fragment libraries (Step 1a). The 180-bp fragments mean that the two paired reads overlap by approximately 20 bp, as are required for assembly by ALLPATHS-LG (Gnerre *et al.* 2011). We also simulated 15× coverage for each parent with 3-kb mate-pair libraries (with 2× 100-bp reads), as well as 10× coverage each of 6-kb mate-pair libraries (also with 2× 100-bp reads; Step 1b). All the data used here has been submitted to the National Center for Biotechnology Information Sequence Read Archive under project accession number SRP031655.

To generate sequences that incorporate known error tendencies of the Illumina technology, reads from all individuals were simulated using the program pIRS (Hu *et al.* 2012). These simulated data include nucleotide errors, indel errors, variance in the mean insert size of paired-end sequences, and the GC-bias in read-depth typical of Illumina data (Hu *et al.* 2012). Although there may be other errors in next-generation sequences not considered here—such as “chimeric” reads from mate-pairs that have incorrectly been put together or adapter sequence contamination—several software packages are available to detect and remove such errors (*e.g.*, MIRA; http://sourceforge.net/projects/mira-assembler/). In comparisons reported herein between the RPGC-produced assembly and the standard assembly, all of these types of errors are of course present (or not) in both datasets.

For our initial genome assembly (Step 2a) we included reads from 10 randomly chosen RILs and both parents, for a total of 60× coverage from the short-insert (180 bp) libraries, 30× coverage from the 3-kb libraries, and 20× coverage from the 6-kb libraries. These sequences were used as input to the ALLPATHS-LG assembler, which was run using default settings. The resulting genome assembly had 1874 contigs contained within 213 scaffolds. Without including gaps, the total length of the assembled genome was 97,561,960 bp; with gaps, it was 99,265,117 bp. By comparison, the most recent assembly of the reference *C. elegans* N2 genome (WBcel215) has a golden path length of 100,286,070 bp. Our initial assembly was used as the framework on which to perform downstream analyses. In addition, we generated a “standard” assembly using reads based only on the N2 parent, including 50× coverage from short-insert libraries, 30× from 3-kb libraries, and 20× from 6-kb libraries. Because these reads are generated from an inbred line—one that presumably will generate fewer errors due to heterozygosity—it can serve as standard against which we compare the RPGC-generated assembly below.

To identify high-quality SNPs and indels (Step 2b), reads from the parents and all of the RILs were mapped against our genome using BWA (v0.6.1; Li and Durbin 2009) with default settings. The raw mapping results were then cleaned and sorted using Picard (v1.77; http://picard.sourceforge.net). We used reads from the same 10 RILs used for genome assembly to make initial variant calls. To minimize the number of false-positive calls caused by misalignment due to nearby indels, the processed mapping results were locally realigned using GATK (v2.34; McKenna *et al.* 2010), and the variants were identified using the GATK UnifiedGenotyper. To ensure call quality, we independently called variants using the GATK HaplotypeCaller and SAMtools (v0.1.17; Li *et al.* 2009). The overlapping set of variants called by all programs was further filtered to include only (i) sites with two alleles, (ii) sites where at least one of the 10 individuals is identified as homozygous for the nonreference base with a minimum of 4× coverage, and (iii) sites where at most one RIL is genotyped as heterozygous (no sites should be heterozygous, apart from those that are due to assembly or sequencing errors). This high-confidence set of variants was then used for base-quality recalibration using GATK, with the default set of covariates. The base-quality recalibrated BAM files were used as input for a second round of variant calling on the same 10 RILs, followed again by the same three filters as described previously. The top 10% of the highest-scoring calls in this set were used as a training set for variant-quality recalibration of the entire set of calls. We then repeated base-quality recalibration and realignment on the whole filtered set of variants on the two parents and all 70 RILs. Finally, we called genotypes for all 70 RILs as well as the two parental genomes using the GATK UnifiedGenotyper (Step 3a). In total, our analysis of the simulated Illumina sequences identified 137,662 SNPs and 20,289 indels. Note that this is more than the simulated number of variants, as both split and merged loci artificially inflate the number of apparent SNPs and indels in our assembly.

We identified candidate alleles that had been erroneously split into separate loci (Step 3b) by first finding duplicated sequences in the genome using the program LASTZ (Harris 2007). Such pairs were further filtered to include only those whose total read-depth was less than 600× (350× was expected for single loci), and those longer than 1000 bp with nucleotide identity >90%. There were 31 such pairs of loci longer than 1000 bp in our initial assembly, with the longest spanning 8946 bp.

For each of the 31 pairs of highly similar loci in the genome, we then asked whether pairs of variants identified at homologous positions within the apparently duplicated pair segregated as indels, with the insertion state in one copy always associated with the deletion state in the other copy within a single individual (Step 4); this is the expected pattern for SNPs at split alleles (Figure 3). In addition to this expected pattern, we also found such variants represented as typical SNPs (and not indels), but with missing genotypes present at one locus instead of a deletion state (Figure S1). Of the initial 31 pairs, 16 had markers within them and could therefore be examined for patterns of inheritance. Of these 16, eight showed genotypes among the recombinants expected for alleles that have been split into multiple loci, and eight showed genotypes expected from two truly paralogous loci. To fix the split alleles for the final assembly (Step 5), we examined the position of the two copies in our initial assembly. Four of the pairs were located adjacent to one another on the same scaffold (sometimes separated by a small gap) and were simply merged together into a single locus. The other four pairs were located on different scaffolds, and we attempted to resolve them based on the linkage map (see below in this section).

We identified paralogous loci that had been erroneously collapsed into a single locus during assembly (Step 4) by finding variants that were genotyped as heterozygous in >90% of RILs. We then asked whether the read-depth around these variants from all RILs together was greater than 650× and less than 1100× and used this information to identify the boundaries of collapsed loci. (We limited the read-depth to less than 1100× to avoid transposable elements, but in principle these could also be disambiguated.) There were 69 such loci longer than 1000 bp in our initial assembly, with the longest spanning 6501 bp. To fix merged loci (Step 5) we examined the mapping of the paired-ends from all three libraries. We expected that merged loci would consist of one duplicate mapped to the correct position in the genome—the one identified with high read-depth and heterozygous sites in the initial assembly—and one that is not in the assembly. To find the location of the unassembled paralog, we looked for paired-end reads with one read mapping to the merged locus and one read mapping to another position in the genome. Manual inspection of the mapping results revealed five merged loci that had multiple reads mapping to a single position near the collapsed locus and to a different scaffold. In all five of these cases, we found a gap at the proposed position where the duplicate locus should have been located in the assembly. Realignment of the sequence from the merged locus with the region surrounding these gaps allowed us to fill in all of them.

We generated a linkage map (Step 6) using the program MSTMap (Wu *et al.* 2008). Markers with identical genotypes across all 70 RILs were collapsed into a single marker in order to reduce redundant genotype information from closely linked sites. To minimize mapping errors due to sequencing errors, we only used variants where at most 5% of the RILs had been genotyped as heterozygotes. We also manually collapsed the genotypes of all split loci identified in Step 4 into a single marker genotype. The final set of markers used to construct the linkage map included 2658 SNPs and indels on 113 of the 213 scaffolds. The minimum number of markers on a single scaffold was 1, and the maximum number was 153. Parameters for MSTMap were adjusted until six major linkage groups were obtained, matching the number of known chromosomes in the *C. elegans* genome. Markers from the six major linkage groups were then run separately in MSTMap to determine the order of markers within each linkage group. The order and orientation of individual scaffolds was resolved using a majority rule in cases where there were discrepancies among markers (scaffolds with only a single markers could not be oriented). In total, at this step we were initially able to place 110 of the 213 scaffolds onto one of the six major linkage groups.

For the four cases in which we detected alleles that had been split into loci on different scaffolds, we examined the mapping of relevant markers in the linkage map in order to determine the correct location of the locus (Step 5). In three of the cases, we were able to find the true location of the single locus. In one case, the two scaffolds mapped to the same location in the genetic map, and closer examination revealed that the two scaffolds could be merged by collapsing the split loci (each of which was located at an end of its scaffold) into a single sequence. In the other two cases one of the loci mapped to the middle of a linkage group, while its paired scaffold did not map at all and appeared to consist of only the split allele. These two mini-scaffolds were removed from the assembly.

We also discovered an unanticipated benefit of the linkage map in improving the assembly. On the basis of the genetic map, we found eight smaller scaffolds varying from 1100 to 3400 bp in length that mapped within larger scaffolds. For seven of these small scaffolds the mapping location appeared to coincide with a gap of approximately the same size within the corresponding larger scaffold. We therefore hypothesized that the small scaffolds could fill the matching gaps, and aligned the smaller scaffold to the region surrounding the gap. In six of the cases the smaller scaffold overlapped sequence flanking the gap, indicating that the shorter sequence was embedded within the longer one. In the last case there was no detectable similarity between the smaller scaffold and flanking sequence, so we inserted it into the middle of the larger corresponding gap with flanking N’s on either side.

### Evaluating assembly accuracy and reconstructing parental genomes

Our final genome consisted of 88 ordered and oriented scaffolds (including 97 of the original 213 scaffolds), comprising 98,533,986 bp (Table 1). Of the remaining 13 small scaffolds with markers, 10 were ordered but not oriented, and 3 had the same position in the linkage map and could not be locally ordered with respect to one another. To assess the accuracy of this genome we determined the fraction of the N2 reference genome covered by correctly ordered and oriented scaffolds. To do this we mapped each scaffold to the reference genome using LASTZ. We found that 100% of our scaffolds were correctly ordered. Similarly, 100% of our scaffolds were correctly oriented; this number does not include the 10 scaffolds that were correctly ordered but that could not be oriented, or the single embedded scaffold that was inserted into a gap without orientation. In total, 96.35% of the N2 genome was covered by the 88 correctly ordered and oriented scaffolds.

**Table 1:** Summary of assemblies

	Standard Assembly	RPGC Assembly
# scaffolds assigned to chromosomes	0*^a^*	110
# scaffolds ordered within chromosomes	0	107
Proportion of scaffolds correctly ordered	N/A	100%
# scaffolds oriented	0	90
Proportion of scaffolds correctly oriented	N/A	100%
Final # scaffolds	236	88
Final total length of assembly (with gaps)	99,320,007 bp	98,533,986 bp

a Scaffolds on the X chromosome could have been assigned based on read-depth if males were sequenced. RPGC, recombinant population genome construction; N/A, not applicable.

These data compare very well to the genome produced by the standard assembly approach, although they may not be typical of every application of RPGC. Notably, our “standard” assembly—which was generated from an inbred line containing no variation—contains a number of collapsed and split loci (Table 2). The split loci are of course 100% identical, but the assembly software has chosen to place them on different scaffolds (in fact, all of these were placed on a mini-scaffold by themselves). In total there are ~13,000 more bases from split loci included in the RPGC assembly, although there are the same number of split loci (Table 2). RPGC was able to correct 95% of these split loci and was also able to confirm that an equal number of highly similar pairs of loci with reduced read-depth were in fact true paralogs (see next paragraph). Collapsed loci could be identified in a standard assembly of an inbred individual by looking for regions that are highly “heterozygous,” but these loci could not be corrected, and in individuals that cannot be inbred these cases cannot be distinguished from native levels of heterozygosity. Overall, we conclude that although sequencing inbred strains undoubtedly has many advantages, sequencing a heterozygous population has not introduced a large amount of error into the assembly and has additionally enabled us to correct or identify many of the assembly errors.

**Table 2:** Assembly error and corrections

	Standard Assembly	RPGC Assembly
# pairs of candidate split loci	N/A	31
# pairs of candidate split loci with markers	N/A	16
# pairs of loci split	9	9
# pairs of loci correctly identified as split	N/A	8
Length of split loci in assembly*^a^*	19,503 bp	32,354 bp
Length of split loci corrected in assembly	0	30,927 bp
# pairs of candidate collapsed loci	N/A	69
# pairs of loci collapsed	44	68
# pairs of loci correctly identified as collapsed	N/A	68
Length of collapsed loci in assembly	73,693 bp	156,468 bp
Length of collapsed loci reassembled in assembly	0	19,505 bp

a The total single-locus length of loci confirmed as split into two loci. RPGC, recombinant population genome construction; N/A, not applicable.

We wished to evaluate the accuracy of the changes we made to the initial assembly on the basis of our analysis of variant segregation patterns among recombinant individuals. These changes are not readily accomplished in standard genome assembly, even with follow-up experiments. For split, collapsed, and embedded loci, we used LASTZ to search our candidate regions against the reference N2 genome (a similar analysis was performed to identify errors in the “standard” assembly). We first confirmed that all seven of the small scaffolds embedded into larger scaffolds were at the correct locations. Also consistent with our predictions, the eight split loci that we identified in our assembly—those with the expected genotypic proportions for split loci—do in fact have only one copy in the N2 reference (Table 2). For all seven of the loci that we were able to merge, we correctly identified the location of the true locus. For the other eight pairs of highly similar loci whose patterns of segregation did not suggest that they were erroneously split, we confirmed that there were two copies in the N2 reference for all of them. Of the 15 highly similar pairs for which we did not have markers, nine had one copy in the reference and six had multiple copies in the reference. Overall, we missed nine pairs of split loci and were not able to correctly identify the true genomic location for another pair that we were able to detect as split.

Of the 69 collapsed loci identified by our analysis, we confirmed that 68 had two or more copies in the reference genome (Table 2); this finding indicates that we have accurately identified such loci. The remaining single collapsed locus yielded fragmented alignments from which it was difficult to determine the correct number of loci in the reference genome. For the five merged loci that we were able to disambiguate and place back into our assembly, comparison with the reference showed that we accurately reassembled the paralog that had been missing into the correct position.

We have likely been able to detect all of the split and collapsed loci in our assembly that are longer than 1000 bp. Even if we conservatively assume that we have detected 50% of such loci, errors of these kinds would still only have affected a very small fraction of the genome. This is likely due to two major factors: the low number of repeats in the *C. elegans* genome and the relatively low level of heterozygosity simulated between the parental strains of the cross. The aim of this idealized test-case was to demonstrate the utility of RPGC, and we do not expect such low numbers of errors in larger, more complex genomes subjected to assembly. Although we were not able to fix all split and collapsed loci detected in our initial assembly, these regions could be subject to further local assembly. Even in cases in which the assembly cannot be improved, variants found within these regions can be marked as likely false positives and can be appropriately handled in follow-up studies.

In addition to constructing a highly accurate reference genome, we wished to know whether we could accurately determine the sequences of the two parental genomes separately. To do this requires that we determine the haplotypic phase of all our varying sites, correctly assigning each allele to the appropriate parental genome. We input the updated genotypes of all 70 RILs and an additional individual purposefully created to be heterozygous at all sites (*i.e.*, an F_1_ individual for the cross described here) into the program BEAGLE (Browning and Browning 2007); markers for each linkage group were run separately for computational efficiency. The haplotypic phase of the completely heterozygous (F_1_) individual is the only one relevant to the analysis, as all of the RILs are homozygous at the vast majority of sites. We were able to determine the phase for 112,310 SNPs in this individual. Of the phased markers, 91.4% were correctly assigned to a haplotype matching the N2 genome. These results also indicate that we are able to infer the genome of the other (N2b) parent using the RPGC approach. No other current genome assembly approach is able to simultaneously reconstruct two phased genomes in a single experiment.

## Discussion

Here we have proposed a new synthetic approach to *de novo* sequence assembly and correction that addresses many of the current challenges of whole-genome sequencing, especially for non-model systems. We evaluated the implementation and accuracy of this proposed methodology by analyzing a simulated recombinant population generated from known sequence data. Our results indicate that RPGC provides a highly accurate method for genome construction, although we have only simulated a single type of recombinant population under a subset of possible conditions. RPGC also provides unprecedented power to reconstruct two phased parental genomes in a single experiment. Nonetheless, the implementation of this method in any specific system will require some careful strategic considerations. We have thus far pointed to several factors that will influence the complexity of genome assembly and construction using RPGC, including the structure and size of the recombinant population, the average number of variants differentiating the parental lines, and the level of heterozygosity expected at each locus. These factors influence the ability to accurately distinguish alleles and paralogs, to accurately and efficiently call genotypes, and to therefore use these data in various steps of assembly and construction within RPGC. Accordingly, they should influence design choices for implementing RPGC in any given system. In the subsections to follow, we discuss two of the most important, and relatively fixed, design considerations: methodological trade-offs and biological constraints.

### Methodological trade-offs

The main trade-offs to consider for implementing RPGC involve balancing experimental size and read-coverage against the accuracy and efficiency of the method. Given a fixed investment in sequencing costs, these trade-offs will influence the selection of the parents to generate recombinant populations and the number of recombinant individuals able to be sequenced.

First, only crosses involving parents that differ at a large enough number of sites will be informative for placing and ordering scaffolds on linkage maps. Because errors at any single SNP may result in incorrect genotypes (see the section *Biological constraints*), arguably the most important factor in this regard is the number of SNPs per assembled scaffold. This quantity can be maximized either by investing in more sequencing (or in a different kind of sequencing) to get longer scaffolds, or by generating or examining recombinant populations from distantly related parents. In the first case, more sequence can be obtained either by adding more coverage per individual to a fixed number of recombinants, or by adding more recombinant individuals; however, sequence read length and coverage can be limited by financial and/or technical limits. Rather than investing in the generation of longer sequences to maximize SNPs/scaffold, an alternative solution for this problem might seem to be selecting parents that are more divergent. However, parents that are too distantly related can produce recombinant populations that suffer from skewed genotypic ratios due to widespread transmission ratio distortion (*e.g.*, Moyle and Graham 2006; Myburg *et al.* 2004; Ross *et al.* 2011; Solignac *et al.* 2004); this compromises the ability to use Mendelian expectations to infer linkage relationships in the recombinant population. Therefore, one design challenge will be identifying experimental parents that differ at a sufficient number of sites, but are not so divergent that their hybrids exhibit substantial levels of marker distortion. Because it is not evident where this point will be in any particular system (*e.g.*, Hall and Willis 2005; Matsubara *et al.* 2011; Payseur and Hoekstra 2005; Zamir and Tadmor 1986), a reasonable compromise might be to choose parents from a single, diverse population or from recently diverged ecotypes. In the latter case, choosing ecotypically diverged parents also allows simultaneous QTL mapping of functional traits that differ between ecotypes, as well as the reconstruction of separate genomes for the two ecotypes. Regardless, because of expected difficulties with sterility and/or marker transmission distortion, parents from different species should generally not be used for RPGC.

Second, for a fixed amount of sequencing investment—ignoring the not insignificant library costs for a moment—there is a trade-off between capturing more meioses (that is, including more recombinant individuals) and more accurately calling genotypes (that is, increasing sequencing coverage of each individual included). The number of recombinant individuals sequenced determines many things, including the ability to order and orient scaffolds within linkage groups and the power to distinguish between alleles and paralogs. In general, an F_2_ population will provide twice as many meioses as a BC_1_ population, with more advanced intercross lines providing more recombination events. Within a chromosome, the number of ordered blocks of scaffolds will also be determined by the number of meioses. Within each such block there will be no information about the ordering or orientation of scaffolds, so increasing the number of meioses has a direct effect on genome construction steps involving the placement and ordering of scaffolds and subsequent QTL mapping resolution.

Nonetheless, with fixed sequencing investment, a larger number of recombinant individuals means lower sequence coverage per individual. Sequence coverage is essential to accurately call genotypes at variable loci—this is the only way to distinguish heterozygotes from homozygotes (recalling that SNPs are initially identified in the total pool of sequences). Without accurate genotype calls alleles cannot be distinguished from paralogs, and constructing accurate linkage maps is made much more difficult. For example, if read-coverage is approximately Poisson-distributed, then with 5× coverage per recombinant we should be able to accurately detect 84% of heterozygous sites in an individual; for 10× coverage, this number jumps to 98% of heterozygous sites. These calculations assume that there is only one SNP per scaffold, and therefore that each genotype call must be accurate. Instead, we can use multiple independent SNPs to bolster our confidence in the genotype of a whole scaffold or, if there is a meiosis within a scaffold, the genotype of each half. The individual genotypes at multiple independent SNPs within a scaffold can therefore be used to better call the genotype of the entire scaffold. Low-coverage sequencing of recombinant populations has already proved successful in organisms with sequenced genomes largely because the location of all markers is known *a priori* in these cases (Huang *et al.* 2009; Xie *et al.* 2010). This allows the use of models that can genotype whole stretches of each chromosome, even when there are less than perfect genotype calls at each marker. For RPGC where there is no information about the relative location of markers along chromosomes, more SNPs per scaffold (up to a limit) will help to accurately call genotypes. With >1 SNP/scaffold, we likely need at least 5× coverage per potentially heterozygous recombinant individual (*e.g.*, F_2_ individual) to accomplish accurate genome construction.

These methodological trade-offs will be necessarily influenced by financial constraints on the design of the experiment. In particular, even when increasing the total number of sequenced recombinants might be preferable, this involves increasing the number of sequencing libraries. Libraries are a fixed, per individual, cost. Given the large amount of DNA sequence produced by a single Illumina HiSeq lane (currently at least dozens of gigabases) for a relatively low price, the costs of library construction for a large recombinant population are expected to rapidly outstrip the sequencing costs. Therefore, increasing per-individual coverage might be more financially feasible in comparison with increasing the total number of recombinant individuals.

Finally, it is worth noting that there are cheaper, alternative approaches to RPGC available if the only aim is to order and orient scaffolds on a map. A number of reduced-representation approaches (*e.g.*, RAD-tags; Baird *et al.* 2008) offer the ability to detect and genotype large numbers of variants more cheaply than whole-genome sequencing. These methods have been previously used to construct linkage maps connecting scaffolds to chromosomes (*e.g.*, Hyten *et al.* 2010) or even individual gene models to chromosomes (*e.g.*, Amores *et al.* 2011). Alternatively, if the average scaffold is long enough and high-molecular-weight DNA is available, then optical mapping also offers the ability to place sequences on chromosomes (*e.g.*, Zhou *et al.* 2009). Both of these approaches avoid the need for a large number of standard barcoded libraries, each of which has a significant cost. However, neither of these approaches has the ability to correct split or collapsed loci either because they do not have dense enough marker information within such loci (RAD-tags) or do not contain genetic information (optical mapping). They therefore are not expected to produce a genome of the same quality as RPGC. By definition, reduced representation approaches also will not encompass as broad a representation of the genome as RPGC.

### Biological constraints

Biological factors such as the reproductive biology and/or evolutionary history of a species can also impose absolute constraints on experimental design choices. Some of these factors (*e.g.*, genome size, generation time, etc.) are outside the control of the researcher and should be considered when identifying an appropriate system for RPGC, rather than when deciding on a population design for the sequencing. However, for several biological factors that rest in the hands of the researcher, the efficiency, accuracy, and success of RPGC can depend on thoughtful design considerations.

Two such factors are the specific identity of the parents of the recombinant population sequenced for RPGC and the genetic structure of the recombinant population itself. Both of these factors will to some extent be constrained by reproductive biology (clutch size, inability to self or inbreed, etc.), and by the evolutionary and breeding history (and therefore genetic characteristics) of the target individuals and the populations from which they are drawn. These constraints and their effects on experimental design can be illustrated by contrasting a relatively simple *vs.* more complex potential scenario when applying RPGC. Under the simplest scenario, the target species is able to self-fertilize (or allows consanguineous brother-sister mating), individual fecundity is high (family sizes can be large), the species segregates few deleterious recessive alleles (inbreeding depression is low), and individuals from different ecotypes are moderately differentiated at the molecular level. This might be the case in species with a history of mixed-mating or biparental inbreeding, and known patterns of local adaptation. For this case the ideal experimental design would use two inbred parents drawn from different locally adapted populations to generate a single F_1_ individual that is selfed to generate a large F_2_ population. This design assures only two alleles per locus in the F_2_s, thereby simplifying both the identification of alleles *vs.* paralogs, and the expected genotypic ratios in the F_2_s at each locus. In addition, large family sizes enable a large F_2_ population to be generated from a single brother-sister F_1_ pair (or selfed F_1_), producing sufficient recombinant individuals to capture enough meioses for genome construction and for QTL mapping. As such, this scenario is potentially the best-case application for RPGC, and closely resembles the *C. elegans* dataset we have simulated here.

Under a more realistic scenario, parents are outbred and F_1_s cannot be self-fertilized, although F_1_ brother-sister mating is possible. In this case, there can be up to four alleles (*i.e.*, haplotypes) segregating at any locus in an F_2_ recombinant population. This will be more challenging for the discrimination of alleles *vs.* paralogs, although in any single F_2_ individual there can be at most only two alleles at each locus, and expected genotypic ratios are still relatively simple (*e.g.*, even with four alleles per locus, there are only 10 possible genotypes and the expected frequency of each homozygote genotype is 1/16). Even so, these challenges are expected to increase rapidly as greater constraints are imposed by the reproductive biology of the target species. For example, if consangineous F_1_ mating is prevented, then four outbred parents will be involved in generating the recombinant population (because F_1_ crosses must be between individuals from two different families), meaning up to eight alleles (haplotypes) and 36 possible genotypes per locus. In this extreme case, each unique homozygous genotype will appear at a frequency of only 1/64.

As these cases illustrate, one of the essential design problems in generating (or identifying existing) populations for RPGC is to minimize the number of alleles segregating per locus—the ideal number is two. The greater the number of segregating alleles, the more sequence coverage is needed to accurate identify each allele and each genotype at a site. The greater the number of individuals contributing alleles to the recombinant population, the larger this problem can become. In some cases, strategic choice of crossing design can reduce some of this dimensionality. For example, in comparison to F_2_s, backcross designs on average reduce by 1/4 the total number of alleles expected to be segregating in a recombinant population. Therefore, backcrossed populations might be preferable in cases where allelic variation is expected to be high.

As an alternative approach to controlled genetic crosses, we can also envision the application of RPGC to systems in which classical genetics is not feasible, although the aforementioned analysis and inference steps will be correspondingly more complex. These cases can use alternative approaches to linkage mapping, or may even take advantage of linkage disequilibrium (LD) in natural populations. For instance, if DNA from a multigeneration pedigree is available, this can be used to a generate linkage map (*e.g.*, Chagné *et al.* 2003). If a large enough number of recombinants is available, linkage maps can be constructed even when only heterozygous parents and offspring are available (*e.g.*, Grattapaglia and Sederoff 1994) or when only a single heterozygous parent and its gametes are available (*e.g.*, Tulsieram *et al.* 1992). Although the distance over which LD extends in natural populations is generally not large enough to aid in constructing a genetic map, certain demographic histories will increase LD tract lengths. A recent study in humans used the long tracts of LD created by population admixture to map the location of dozens of unplaced scaffolds in the genome (Genovese *et al.* 2013). If similar populations are available in other species, patterns of LD in nature could be used in an RPGC-like approach.

Finally, other biological factors also will shape experimental design decisions by imposing logistical constraints. For example, organismal body size imposes constraints on the amount of DNA available per individual; given current technologies, small organisms (*e.g.*, nematodes, flies, etc.) are unlikely to have sufficient high-quality DNA per individual to allow sequencing, especially if the goal is to perform simultaneous trait analysis on the same genotypes. For these organisms it would be necessary to use alternative (non-F_2_) population structures—such as RILs—that enable replicate homozygous recombinant genotypes and therefore allow both destructive sampling to obtain DNA and simultaneous phenotype analysis within each line.

Given these biological realities, some systems will clearly be better targets for RPGC than others. However, it should be clear that many complex scenarios can be accommodated with appropriate design choices during the selection of population parents and the generation or identification of the recombinant population. This includes cases where inbred lines cannot be generated and outcrossing is enforced, or even cases in which individuals are sampled from nature. Although these cases might place specific bounds on the efficiency and accuracy of individual steps in the assembly, genome construction, and mapping process, they are not absolute barriers to using the RPGC approach. This makes RPGC especially relevant to non-model systems that are poorly served by current whole-genome sequencing approaches.

## Conclusions

Here we have proposed an approach that resolves three critical problems in applying NGS technology to generating *de novo* genomes, especially in traditionally nonmodel systems: first, genome assembly problems, including resolving alleles *vs.* paralogs, and identifying collapsed paralogs; second, genome construction problems, including the identification of linkage groups and the placement and ordering of scaffolds onto chromosomes; and third, limitations on immediate applications, including the absence of SNPs and the inability to conduct quantitative trait mapping. Together, these factors currently conspire to place whole-genome sequencing beyond the reach of many species that are rich in organismal (*e.g.*, physiological, behavioral, and ecological) data. Although recombinant populations have been used to assign scaffolds to chromosomes in multiple genome projects, RPGC collapses all three phases of the transition from genomic nonmodel to model organism (genome sequencing, linkage map construction, application) into a single experiment, and solves problems of genome assembly that are not addressed by typical linkage mapping approaches.

We have applied RPGC to a simulated recombinant population, one that is idealized in many respects. It will almost surely be the case that when applied to real sequence data (possibly containing errors not included in our simulated reads) and real recombinant populations (containing, for instance, residual heterozygosity not simulated here), the genome produced will not reach 96% accuracy. However, we also have not designed RPGC with a single sequencing platform, assembly algorithm, read-mapping software package, or SNP-calling tool in mind; our approach can be used with any combination of programs, and can therefore take advantage of all future improvements in any of these steps.

It is important to note that RPGC does not solve all of the problems of genome construction, including sequencing errors (*e.g.*, Ye *et al.* 2011) or highly fragmented scaffolds and contigs (*e.g.*, Mortazavi *et al.* 2010). It is also not ideal for organisms in which controlled genetic crosses are not possible. However, the flexibility of the method in terms of both choices in sequencing technology and assembly tools means that the general approach will be feasible and economical for the foreseeable future. Moreover, because RPGC is able to accommodate and/or correct for the challenges that frequently emerge for nonmodel systems, implementing this method in a range of empirical systems is expected to be more successful than traditional genome assembly processes.

## Supplementary Material

Supporting Information
