# Supplementary material for: Sequencing, Assembling, and Correcting Draft Genomes Using Recombinant Populations
Source: G3 (Bethesda). 2014 Feb 13;4(4):669–79. doi: 10.1534/g3.114.010264 (PMC4059239; doi:10.1534/g3.114.010264)
Supplement: Supporting Information [file supp_4_4_669__index.html]

Sequencing, Assembling, and Correcting Draft Genomes Using Recombinant Populations — Supporting Information 

# Sequencing, Assembling, and Correcting Draft Genomes Using Recombinant Populations

## Supporting Information for Hahn, Zhang, and Moyle, 2014

**Files in this Data Supplement:**

- Figure S1 - Alternate pattern of segregation of split alleles through an F2 cross. (PDF, 125 KB)
- File S1 - RPGC Manual (PDF, 1 MB)
