# Supplementary material for: Sequencing, Assembling, and Correcting Draft Genomes Using Recombinant Populations
Source: G3 (Bethesda). 2014 Feb 13;4(4):669–79. doi: 10.1534/g3.114.010264 (PMC4059239; doi:10.1534/g3.114.010264)
Supplement: Supporting Information [file supp_g3.114.010264_010264SI.pdf]

G3 · Genes | Genomes | Genetics

Sequencing, assembling, and correcting draft genomes using recombinant populations

Matthew W. Hahn[*,§,1], Simo V. Zhang[§], and Leonie C. Moyle[*]

[*]Department of Biology and [§]School of Informatics and Computing, Indiana University, Bloomington, IN 47405

[1]Corresponding author. 1001 E. 3rd St., Indiana University, Bloomington, IN 47405. E-mail: mwh@indiana.edu, Phone: (812)856-7001.

DOI: 10.1534/g3.114.010264

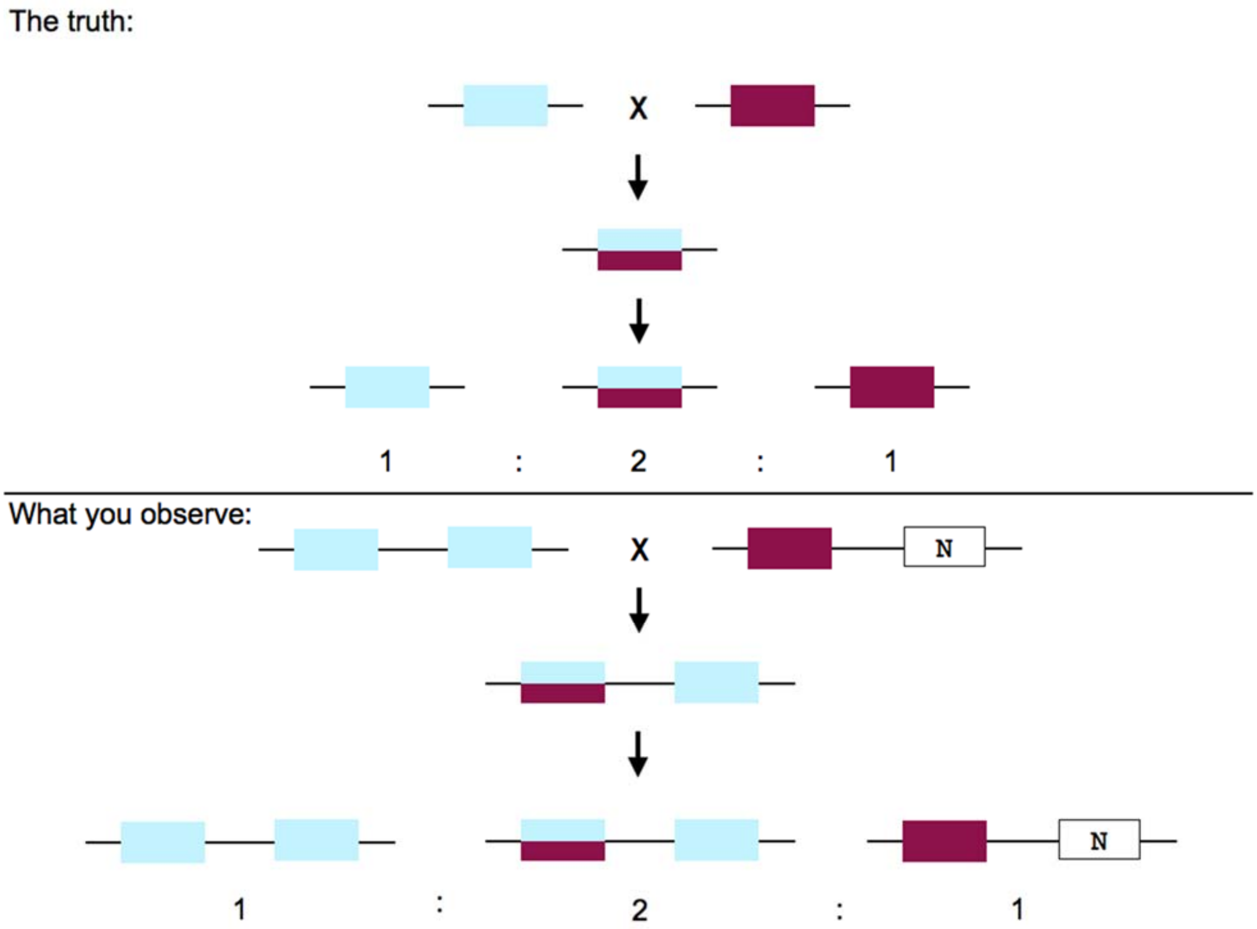

Figure S1 Alternate pattern of segregation of split alleles through an F_2 cross. The top panel shows the physical reality of a single gene that differs in allelic sequence between the parents. The F_1 and half the F_2s are heterozygous. The bottom panel shows how, when alleles are split into two loci, one locus retains the allelic variation while the other contains only one allele (i.e. does not show a SNP). In individuals that have the blue allele at the first locus, the second locus also shows the blue allele. In individuals homozygous for the red allele, however, the second locus shows missing data at the corresponding site. Note that the pattern of segregation at the first locus is as expected.

File S1

RPGC Manual

Available for download as a PDF file at http://www.g3journal.org/lookup/suppl/doi:10.1534/g3.114.010264/-/DC1
