# Supplementary material for: Sequencing, Assembling, and Correcting Draft Genomes Using Recombinant Populations
Source: G3 (Bethesda). 2014 Feb 13;4(4):669–79. doi: 10.1534/g3.114.010264 (PMC4059239; doi:10.1534/g3.114.010264)
Supplement: Supporting Information [file supp_g3.114.010264_FileS1.pdf]

RPGC Manual

Introduction

Here we present a new approach for producing *de novo* whole genome sequences--recombinant population genome construction (RPGC)--that solves many of the problems encountered in standard genome assembly (see more details in the accompanying manuscript). This manual goes through the analysis explained in the manuscript with great detail.

Requirements

To run our RPGC walk-through below, you will need the following software installed:

- pIRs v1.10
- ALLPATHS-LG build 41292
- BWA v0.6.1
- Picard v1.77
- GATK v.2.3.4
- SAMtools v0.1.18
- LASTZ 1.02.00
- MSTMap
- BEAGLE v3.3.2
- JAVA 1.6.0_26

You will also need python 2.7 or above to run our home-brew python scripts.

Note that RPGC does not require any particular software at each step. The ones listed above best served our interests; e.g. you could use SOAPdenovo instead of ALLPATHS-LG to generate your assemblies. The general approach remains the same.

Conventions

The following coventions are used in this manual:

1. Command-line arguments are split into separate lines for clarity.

   For example:

   ```
   pirs diploid -i n2.fasta -s 0.001 -d 0.0001 -v 0.000001 -o n2b.fasta
   ```

   becomes

   ```
   pirs diploid -i n2.fasta \
   -s 0.001 -d 0.0001 -v 0.000001 \
   -o n2b.fasta
   ```

2. Command-line settings shown in red use our home-brew python scripts.

3. Programs within square brackets indicate that valid paths to where the programs are installed are required. In the example below, you need to specify the path to the directory where pirs can be found.

   ```
   [pirs] diploid -i n2.fasta \
   -s 0.001 -d 0.0001 -v 0.000001 \
   -o n2b.fasta
   ```

4. Command-line arguments within <> indicate valid paths to where files or directories are stored are expected. In the example below, you need to provide both a path to the n2.fasta file and a path to the output.

```
[pirs] diploid -i <n2.fasta> \
-s 0.001 -d 0.0001 -v 0.000001 \
-o <n2b.fasta>
```

5. Paired-end fastq files. So that we do not need to list every single file, we use the following shorthand:

```
rils1_5x_180_1.fastq
rils1_5x_180_2.fastq
```

will be abbreviated as

```
rils1_5x_180_*.fastq
```

Getting Help

RPGC uses both external programs and our home-made python scripts. For our python scripts, you can always call -h for help .

For example:

python scripts.py -h

As for the external programs, we provide command-line settings we used in our analysis. You can always customize them based on your needs.

If you have further questions, please email either: Matthew Hahn (mwh@indiana.edu) or Simo Zhang (simozhan@indiana.edu). All of the scripts and sample data files can be found here:

http://sites.bio.indiana.edu/~hahnlab/RPGC.html

Environment

All the commands were tested successfully on a 64-bit Red Hat Linux server at Indiana University.

What follows is a step-by-step description of how we simulated data, and how we used RPGC to analyze that data.

Simulating A Recombinant Population

We used the reference *C. elegans* strain N2 as one parental genome, and simulated a second parent, N2b, using pIRS (v1.10).

Input:
n2.fasta
Output:
n2b.fasta

```
[pirs] diploid \
-i <n2.fasta> \
-s 0.001 -d 0.0001 -v 0.000001 \
-o <n2b.fasta>
```

We simulated 70 individual RILs equivalent to eight generations of selfing by a set of individual F2s that were produced from crossing perfectly isogenic parental strains and self-fertilizing the resulting F1. We assumed that this resulted in a total of five crossing-over events per chromosome in the sequenced RILs, and that recombination events were uniformally distributed along chromosomes.

Input:
n2.fasta
n2b.fasta
Output:
rils1_m.fasta, rils1_p.fasta
rils2_m.fasta, rils2_p.fasta
...
rils70_m.fasta rils70_p.fasta

```
python [sim_recombinants.py] \
-cross_design RILs \
-p1 <n2b.fasta> -p2 <n2.fasta> \
-popsize 70 -ncross 5 \
-o <rils>
```

Sequencing Recombinants

For 70 RILs and the two parental strains, we simulated 5X sequence coverage of 2*100 bp paired-end Illumina reads from 180 bp fragment libraries using pIRS (v1.10). Command-line settings for generating reads from n2, n2b and rils1 are shown, as all the other 69 RILs will be applied with the same settings.

Input:

n2.fasta
n2b.fasta
rils1_m.fasta rils1_p.fasta
rils2_m.fasta rils2_p.fasta
...
rils70_m.fasta rils70_p.fasta

Output:

n2_5x_180_*.fastq
n2b_5x_180_*.fastq
rils1_5x_180_*.fastq
rils2_5x_180_*.fastq
...
rils70_5x_180_*.fastq

```
[pirs] simulate \
-i <n2.fasta> -I <n2.fasta> \
-x 5.0 -m 180 -v 5 -e 0.01 \
-o <n2_5x_180>

[pirs] simulate \
-i <n2b.fasta> -I <n2b>.fasta \
-x 5.0 -m 180 -v 5 -e 0.01 \
-o <n2b_5x_180>

[pirs] simulate \
-i <rils1_m.fasta> -I <rils1_p.fasta> \
-x 5.0 -m 180 -v 5 -e 0.01 \
-o <rils1_5x_180>
```

Sequencing Recombinants

We also simulated 15X coverage for each parent with 3 Kb mate-pair libraries (with 2*100 bp reads), as well as 10X coverage each of 6 Kb mate-pair libraries (also with 2*100 bp reads)

Input:

n2.fasta
n2b.fasta

Output:

n2_15x_3000_*.fastq
n2b_15x_3000_*.fastq
n2_10x_6000_*.fastq
n2b_10x_6000_*.fastq

```
[pirs] simulate \
-i <n2.fasta> -I <n2.fasta> \
-x 15.0 -m 3000 -v 300 -e 0.01 \
-o <n2_15x_3000>
```

```
[pirs] simulate \
-i <n2.fasta> -I <n2.fasta> \
-x 10.0 -m 6000 -v 600 -e 0.01 \
-o <n2_10x_6000>
```

```
[pirs] simulate \
-i <n2b.fasta> -I <n2b.fasta> \
-x 15.0 -m 3000 -v 300 -e 0.01 \
-o <n2b_15x_3000>
```

```
[pirs] simulate \
-i <n2b.fasta> -I <n2b.fasta> \
-x 10.0 -m 6000 -v 600 -e 0.01 \
-o <n2b_10x_6000>
```

Genome Assembly

We built a *de novo* genome assembly using ALLPATHS-LG (build 41292). We first prepared the data as ALLPATHS-LG suggests. Reads from two parents plus 10 randomly chosen RILs were used.

Input:

n2_5x_180_*.fastq
n2_15x_3000_*.fastq
n2_10x_6000_*.fastq
n2b_5x_180_*.fastq
n2b_15x_3000_*.fastq
n2b_10x_6000_*.fastq
rils1_5x_180_*.fastq
...
rils10_5x_180_*.fastq

Output:

assembled_genome.fasta

```
[PrepareAllPathsInputs.pl] \
DATA_SUBDIR=<frag_60x_short_50x> \
PICARD_TOOLS_DIR=[picard-tools-1.77] \
IN_GROUPS_CSV=<in_groups.csv> \
IN_LIBS_CSV=<in_libs.csv> \
PLOIDY=2 PHRED_64=True \
OVERWRITE=True

[RunAllPathsLG] PRE=<rpgc_assembly> \
DATA_SUBDIR=<frag_60x_short_50x> \
RUN=run1 \
SUBDIR=subdir \
OVERWRITE=True \
```

The “in_groups.csv” and “in_libs.csv” files are provided along with our python scripts.

Read Mapping

Reads from the parents and all of the RILs were mapped against our assembled genome using BWA (v0.6.1) with default settings. Here you can decide to either run BWA (or your mapper of choice) on each of the individuals separately, or to use our home-brew python wrapper script that calls BWA internally. But before making up your mind, you need first to index your assembled genome using the index subprogram built in BWA.

Input:
  assembled_genome.fasta
Output:
  assembled_genome

```
[bwa] index \
-p <assembled_genome> \
<assembled_genome.fasta>
```

The command-line for using our python script is shown below. Mapping results will be organized by individual under specified "mapping_dir".

Input:
  configuration file
  assembled_genome
Output:
  SAM files

```
python [reads_mapping_runner.py] \
-config <bwa.config> \
-db <assembled_genome> \
-t 8 \
-outdir <mapping_dir>
```

Read Mapping

Because we use paired-end read mapping, two subprograms in BWA are called internally from our python script: aln and sampe, using the default settings. Below is an example of our script calling BWA on one of the RILS.

```
[bwa] aln -t 8 <assembled_genome> \
<rils1_5x_180_1.fastq>
> <rils1_5x_180_1.sai>

[bwa] aln -t 8 <assembled_genome> \
<rils1_5x_180_2.fastq>
 > <rils1_5x_180_2.sai>

[bwa] sampe <assembled_genome> \
<rils1_5x_180_1.sai> <rils1_5x_180_2.sai> \
<rils1_5x_180_1.fastq> <rils1_5x_180_2.fastq> \
> <rils1_5x_180.sam>
```

Currently, our python script only supports calling BWA with default setting. Only the number of threads can be customized through our python script command-line (-t option).

Read Mapping

To run our python script, you have to provide a configuration file whose format is defined as follows:

1. one individual per line
2. each line consists of three space-separated columns:
    - (i). individual ID
    - (ii). fastq file of one end of the read
    - (iii). fastq file of the other end of the read
    - (iv). fastq file of single-end reads if any (Optional)
3. lines starting with "#" will be taken as comments and will not be read by the script

An example of configuration file, bwa.config, will be provided along with our python scripts.

After Read Mapping

The raw mapping results in SAM format are further processed using Picard (v1.77). Similar to what we did for read mapping, we used another home-brew python wrapper script to call Picard internally with its built-in subprograms:

1. CleanSam.jar
2. SamFormatConverter.jar
3. SortSam.jar
4. MarkDuplicates.jar
5. AddOrReplaceReadGroups.jar
6. MergeSamFiles.jar

Our python script works by first parsing the configuration file (see more details below) to get the SAM file and read group definition for each individual. Then it runs Picard subprograms 1-5 above on each individual SAM file. Lastly, it merges all individually processed BAM files into one BAM file that is ready for variant calling. Command-line for using our python script is shown below.

Input:
- configuration file
- path to where Picard installed

Output:
- BAM file prefixed by "indvs"
- log file from Picard

```
python [post_mapping_pipeline.py] \
-config <picard.config> \
-picard <picard-tools-1.77> \
-p <indvs> \
-picard_log <picard.log>
```

After Read Mapping

The command-line settings of the Picard subprograms running on one of the individuals are shown below. The same settings are applied to the SAM files of other individuals. Paths to inputs and outputs within each step are handled internally by our python script.

```
java -Xmx2g -jar CleanSam.jar \
I=rils1_5x_180.sam O=rils1_5x_180.cleaned.sam \
VALIDATION_STRINGENCY=STRICT

java -Xmx2g -jar SamFormatConverter.jar
I=rils1_5x_180.cleaned.sam \
O=rils1_5x_180.cleaned.bam \
VALIDATION_STRINGENCY=LENIENT

java -Xmx2g -jar SortSam.jar \
I=rils1_5x_180.cleaned.bam \
O=rils1_5x_180.cleaned.sort.bam \
SO=coordinate \
VALIDATION_STRINGENCY=LENIENT
```

After Read Mapping

```
java -Xmx2g -jar MarkDuplicates.jar \
REMOVE_DUPLICATES=true \
I=rils1_5x_180.cleaned.sort.bam \
O=rils1_5x_180.cleaned.sorted.rmdup.bam \
M=dup.report \
VALIDATION_STRINGENCY=LENIENTS

java -Xmx2g -jar AddOrReplaceReadGroups.jar \
I=rils1_5x_180.cleaned.sorted.rmdup.bam\
O=rils1_5x_180.cleaned.sort.rmdup.rg.bam \
SO=coordinate \
ID=rils1 SM=rils1 PU=run LB=frag PL=Illumina
```

Individually processed BAM files are then merged into one BAM file using the following command-line setting.

```
java -Xmx2g -jar MergeSamFiles.jar \
I=rils1_5x_180.cleaned.sorted.rmdup.rg.bam \
I= ... I= ... I=... \
O=indvs.gatk_ready.bam \
SO=coordinate CREATE_INDEX=true \
USE_THREADING=true \
VALIDATION_STRINGENCY=LENIENT
```

After Read Mapping

We divided the entire dataset (70 RILs and two parents) into the following two groups and ran our python script separately:

1. SAM files of the same 10 RILs used in genome assembly
2. SAM files of all the other individuals

We then get two BAM files that were used as inputs to variant calling:

1. indvs.ready.bam
2. all_indvs.ready.bam (merged from all BAM files of 70 RILs and 2 parents)

To run our python script, you are required to provide a configuration file whose format is defined as follows:

1. one individual per line
2. each line consists of three space-separated columns:
    (i). read group definition
    (ii) SAM file from pair-end mapping
    (iii) SAM file from single-end mapping if any (Optional)
3. lines starting with "#" will be taken as comments and will not be read by the script

An example of configuration file, picard.config, will be provided along with our python scripts.

Before Initial Variant Calling

We used a processed BAM file from the same 10 RILs used for genome assembly to make initial variant calls using GATK (v2.3.4):

1. RealignerTargetCreator
2. IndelRealigner

Input:

indvs.ready.bam

assembled_genome.fasta

Intermediate:

indvs.initial.interval

Output:

indvs.gatk.realigned.bam

```
java [GenomeAnalysisTK.jar] \
-T RealignerTargetCreator \
-I  <indvs.ready.bam> \
-R <assembled_genome.fasta> \
-fixMisencodedQuals \
-o <indvs.initial.interval>
```

```
java [GenomeAnalysisTK.jar] \
-T IndelRealigner \
-I  <indvs.gatk_ready.bam> \
-R <assembled_genome.fasta> \
-fixMisencodedQuals \
-targetIntervals <indvs.initial.interval> \
--consensusDeterminationModel USE_SW \
-o <indvs.gatk.realigned.bam>
```

Initial Variant Calling

We then used GATK UnifiedGenotyper on the realigned BAM file to make the initial call set. Note that because we did not have a set of known variants, we could not run base quality recalibration at this stage.

Input:
indvs.gatk.realigned.bam
assembled_genome.fasta
Output:
indvs.gatk.ug.initial.vcf

```
java [GenomeAnalysisTK.jar] \
-T UnifiedGenotyper\
-I  <indvs.gatk.realigned.bam> \
-R <assembled_genome.fasta> \
-glm BOTH \
--genotyping_mode Discovery  -nt 4 \
-dcov 50 \
-stand_emit_conf 30 -stand_call_conf 10 \
-o <indvs.gatk.ug.initial.vcf>
```

To ensure call quality, we independently used SAMtools (v0.1.18) and the GATK HaplotyeCaller .

Input:
indvs.gatk.realigned.bam
assembled_genome.fasta
Output:
indvs.samtools.initial.vcf

```
[samtools] mpileup -d 50 \
<assembled_genome.fasta> \
<indvs.gatk.realigned.bam> | \
[bcftools] view -bvcg - \
> <indvs.samtools.raw.bcf>

[bcftools] view <indvs.samtools.raw.bcf> \
> <indvs.samtools.initial.vcf>
```

Initial Variant Calling

Input:

indvs.gatk.realigned.bam
assembled_genome.fasta

Output:

indvs.gatk.hc.initial.vcf

```
java [GenomeAnalysisTK.jar] \
-T HaplotypeCaller \
-I  <indvs.gatk.realigned.bam> \
-R <assembled_genome.fasta> \
--genotyping_mode Discovery \
-dcov 50 \
-stand_emit_conf 30 -stand_call_conf 10 \
--out <indvs.gatk.hc.initial.vcf>
```

After Initial Call and Before Second Call

We took the overlapping set of variants made by all three programs using GATK SelectVariants.

Input:

indvs.gatk.ug.initial.vcf
indvs.gatk.hc.initial.vcf
assembled_genome.fasta

Output:

indvs.gatk.ug_hc.initial.vcf

```
java -jar [GenomeAnalysisTK.jar] \
-T SelectVariants \
-R <assembled_genome.fasta> \
--variant <indvs.gatk.ug.initial.vcf> \
--concordant <indvs.gatk.hc.initial.vcf> \
-o <indvs.gatk.ug_hc.initial.vcf>
```

After Initial Call and Before Second Call

Input:
indvs.gatk.ug_hc.initial.vcf
indvs.samtools.initial.vcf
assembled_genome.fasta
Output:
indvs.gatk.ug_hc_smt.initial.vcf

```
java -jar [GenomeAnalysisTK.jar] \
-T SelectVariants \
-R <assembled_genome.fasta> \
--variant <indvs.gatk.ug_hc.initial.vcf> \
--concordant <indvs.samtools.initial.vcf> \
-o <indvs.gatk.ug_hc_smt.initial.vcf>
```

The overlapping call set was then further filtered to include only i) sites with two alleles, ii) sites where at least one of the 10 individuals is identified as homozygous for the non-reference base with a minimum of 4X coverage, and iii) sites where at most one RIL is genotyped as heterozygous. Below, we first filtered sites with more than two alleles using GATK SelectVariants.

Input:
indvs.gatk.ug_hc_smt.initial.vcf
assembled_genome.fasta
Output:
indvs.gatk.ug_hc_smt.initial.bi.vcf

```
java -jar [GenomeAnalysisTK.jar] \
-T SelectVariants \
-R <assembled_genome.fasta> \
--variant <indvs.gatk.ug_hc_smt.initial.vcf> \
-restrictAllelesTo BIALLELIC \
-o <indvs.gatk.ug_hc_smt.initial.bi.vcf>
```

After Initial Call and Before Second Call

Then we used our home-made python script to further filter the biallelic call-set based on the second and third rules. In cases where different thresholds defined in rule 2 and 3 are required, you can customize them through -filter option as long as its defined format is followed.

Input:
indvs.gatk.ug_hc_smt.initial.bi.vcf
Output:
indvs.high_conf.initial.vcf

```
python [variant_calls_filter.py] \
-raw_vcf <indvs.gatk.ug_hc_smt.initial.bi.vcf> \
-filtered_vcf <indvs.high_conf.initial.vcf> \
-filter 1:4:1
```

After Initial Call and Before Second Call

The filtered call-set was then treated as a high-confidence set of variants and used for base re-calibration (BQSR) using GATK, with the default set of covariates.

Input:
- indvs.gatk.realigned.bam
- assembled_genome.fasta
- indvs.high_conf.initial.vcf

Intermediate:
- indvs.gatk.bqsr.grp

Output:
- indvs.gatk.realigned.bqsr.bam

```
java -jar [GenomeAnalysisTK.jar] \
-T BaseRecalibrator \
-R <assembled_genome.fasta> \
-I <indvs.gatk.realigned.bam> \
--knownSites <indvs.high_conf.initial.vcf> \
-o <indvs.gatk.bqsr.grp>

java -jar [GenomeAnalysisTK.jar] \
-T PrintReads \
-I <indvs.gatk.realigned.bam> \
-R <assembled_genome.fasta> \
-BQSR <indvs.gatk.bqsr.grp> \
-o <indvs.gatk.realigned.bqsr.bam>
```

Second-Round VariantsCalling

We then used the base-quality recalibrated BAM file as input for a second round of variant calling using GATK UnifiedGenotyper.

Input:

indvs.gatk.realigned.bqsr.bam
assembled_genome.fasta

Output:

indvs.gatk.ug.second.vcf

```
java [GenomeAnalysisTK.jar] \
-T UnifiedGenotyper\
-I  <indvs.gatk.realigned.bqsr.bam> \
-R <assembled_genome.fasta> \
-glm BOTH \
--genotyping_mode Discovery  -nt 4 \
-dcov 50 \
-stand_emit_conf 30 -stand_call_conf 10 \
-o <indvs.gatk.ug.second.vcf>
```

After Second-Round Call

We first applied the same three filters as we did for the first call set.

Input:
indvs.gatk.ug.second.vcf
assembled_genome.fasta
Intermediate:
indvs.gatk.ug.second.bi.vcf
Output:
indvs.gatk.ug.second.bi.filtered.vcf

```
java -jar [GenomeAnalysisTK.jar] \
-T SelectVariants \
-R <assembled_genome.fasta> \
--variant <indvs.gatk.ug.second.vcf> \
-restrictAllelesTo BIALLELIC \
-o <indvs.gatk.ug.second.bi.vcf>

python [variant_calls_filter.py] \
-raw_vcf <indvs.gatk.ug.second.bi.vcf> \
-filtered_vcf <indvs.gatk.ug.second.bi.filtered.vcf> \
-filter 1:4:1
```

In addition, we extracted the top 10% of the highest-scoring calls in the resulting set as a training set for variant-quality recalibration (VQSR) of the entire set of calls, using a home-made python script shown below. The value for the percentage of top highest-scoring calls is customizable through "-top_percent" option.

After Second-Round Call

Input:

indvs.gatk.ug.initial.bi.filtered.vcf

Output:

indvs.gatk.ug.train.snps.vcf
indvs.gatk.ug.train.indels.vcf

```
python [get_training_variants.py] \
-vcf <indvs.gatk.ug.second.bi.filtered.vcf> \
-out <indvs.gatk.ug.train> \
-top_percent 10
```

Variant Recalibration

As GATK suggests on their website, the VQSR was done for SNPs and indels separately because they were called by UnifiedGenotyper.

Input:

indvs.gatk.ug.train.snps.vcf
assembled_genome.fasta
indvs.gatk.second.vcf

Output:

indvs.gatk.vqsr.snps.recal
indvs.gatk.vqsr.snps.tranche
indvs.gatk.vqsr.snps.plots

```
java -jar [GenomeAnalysisTK.jar] \
-T  VariantRecalibrator \
-R <assembled_genome.fasta> \
-input <indvs.gatk.second.vcf> \
-an QD -an DP -an FS -an MQRankSum \
-an ReadPosRankSum \
-mode SNP \
-resource:highscoreset,known=true,training=true,truth=true,prior=10.0 <indvs.gatk.ug.train.snps.vcf> \
-recalFile <indvs.gatk.vqsr.snps.recal> \
-tranchesFile <indvs.gatk.vqsr.snps..tranche> \
-rscriptFile <indvs.gatk.vqsr.snps.plots>
```

Variant Recalibration

Input:

indvs.gatk.ug.train.indels.vcf
assembled_genome.fasta
indvs.gatk.second.vcf

Output:

indvs.gatk.vqsr.indels.recal
indvs.gatk.vqsr.indels.tranche
indvs.gatk.vqsr.indels.plots

```
java -jar [GenomeAnalysisTK.jar] \
-T  VariantRecalibrator \
-R <assembled_genome.fasta> \
-input <indvs.gatk.second.vcf> \
-an QD -an FS -an HaplotypeScore \
-an ReadPosRankSum \
-mode INDEL \
-resource:highscoreset,known=true,train-
ing=true,truth=true,prior=10.0 <indvs.gat-
k.ug.train.indels.vcf> \
-recalFile <indvs.gatk.vqsr.indels.recal> \
-tranchesFile <indvs.gatk.vqsr.indels..tranche> \
-rscriptFile <indvs.gatk.vqsr.indels.plots>
```

For both SNP and indel recalibration, we tried different annotation combinations ("-an" option) and picked the one that best trained the mixture Gaussian models based on the .recal, .tranche, and .plots files. Also from the .tranche file you can decide at what threshold level (specified by "-ts_filter_level" in ApplyRecalibration command-line setting below) you want to filter out your calls.

Then we applied the best SNP and indel model, respectively, on our second call-set, using the GATK ApplyRecalibration.

Variant Recalibration

Input:

indvs.gatk.vqsr.snps.recal
indvs.gatk.vqsr.snps.tranche
indvs.gatk.vqsr.snps.plots
assembled_genome.fasta
indvs.gatk.second.vcf

Output:

indvs.gatk.snps.final.vcf

```
java -jar [GenomeAnalysisTK.jar] \
-T  ApplyRecalibration \
-R <assembled_genome.fasta> \
-input <indvs.gatk.ug.second.vcf> \
-tranchesFile <indvs.gatk.vqsr.snps.tranche> \
-recalFile <indvs.gatk.vqsr.snps.recal> \
-mode SNP --ts_filter_level 99.95 \
-o <indvs.gatk.snps.final.vcf>
```

Input:

indvs.gatk.vqsr.indels.recal
indvs.gatk.vqsr.indels.tranche
indvs.gatk.vqsr.indels.plots
assembled_genome.fasta
indvs.gatk.second.vcf

Output:

indvs.gatk.indels.final.vcf

```
java -jar [GenomeAnalysisTK.jar] \
-T  ApplyRecalibration \
-R <assembled_genome.fasta> \
-input <indvs.gatk.ug.second.vcf> \
-tranchesFile <indvs.gatk.vqsr.indels.tranche> \
-recalFile <indvs.gatk.vqsr.indels.recal> \
-mode INDEL --ts_filter_level 95 \
-o <indvs.gatk.indels.final.vcf>
```

Variant Recalibration

We then combined the recalibrated SNP and indel calls to make the final complete call-set.

Input:

indvs.gatk.snps.final.vcf
indvs.gatk.indels.final.vcf
assembled_genome.fasta

Output:

indvs.gatk.final.vcf

```
java -jar [GenomeAnalysisTK.jar] \
-T CombineVariants \
-R <assembled_genome.fasta> \
--variant <indvs.gatk.snps.final.vcf> \
--variant <indvs.gatk.indels.final.vcf> \
-o <indvs.gatk.final.vcf>
```

Before Genotyping

Within our final call-set, there were some variant sites that had both a SNP and indel called. To make things simpler, we removed sites where either both calls failed or passed VQSR using our home-brew python script shown below.

Input:

indvs.gatk.final.vcf

Output:

indvs.gatk.final.rmdup.vcf
targets.report

```
python [fix_redundant_calls.py] \
-vcf <indvs.gatk.final.vcf> \
-targets <targets.report> \
-out <indvs.gatk.final.rmdup.vcf>
```

Before Genotyping

We further generated a call-set with only calls passing the VQSR filter (in VCF file the FILTER fields are all PASS), and used it as the known variant input to realignment and BQSR of the BAM file of all 70 RILs and two parents.

Input:
indvs.gatk.final.rmdup.vcf
assembled_genome.fasta
Output:
indvs.gatk.final.pass.rmdup.vcf

```
java -jar [GenomeAnalysisTK.jar] \
-T SelectVariants \
-R <assembled_genome.fasta> \
--variant <indvs.gatk.final.rmdup.vcf> \
--excludeFiltered \
-o <indvx.gatk.final.pass.rmdup.vcf>
```

We then repeated base-quality recalibration and realignment on the two parents and all 70 RILs using the final call-set as known.

Before Genotyping

Input:

all_indvs.ready.bam
indvs.gatk.final.pass.rmdup.vcf
assembled_genome.fasta

Intermediate:

all_indvs.initial.interval

Output:

all_indvs.gatk.realigned.bam

```
java [GenomeAnalysisTK.jar] \
-T RealignerTargetCreator \
-I  <all_indvs.ready.bam> \
-R <assembled_genome.fasta> \
--known <indvs.gatk.final.pass.rmdup.vcf> \
-fixMisencodedQuals \
-o <all_indvs.initial.interval>

java [GenomeAnalysisTK.jar] \
-T IndelRealigner \
-I  <all_indvs.ready.bam> \
-R <assembled_genome.fasta> \
--knownAlleles <indvs.gatk.final.pass.rmdup.vcf> \
-fixMisencodedQuals \
-targetIntervals <indvs.initial.interval> \
--consensusDeterminationModel USE_SW \
-o <all_indvs.gatk.realigned.bam>
```

Before Genotyping

Input:

all_indvs.gatk.realigned.bam
indvs.gatk.final.pass.rmdup.vcf
assembled_genome.fasta

Intermediate:

all_indvs.gatk.bqsr.grp

Output:

all_indvs.gatk.realigned.bqsr.bam

```
java -jar [GenomeAnalysisTK.jar] \
-T BaseRecalibrator \
-R <assembled_genome.fasta> \
-I <all_indvs.gatk.realigned.bam> \
--knownSites <indvs.gatk.final.pass.rmdup.vcf> \
-o <all_indvs.gatk.bqsr.grp>
```

```
java -jar [GenomeAnalysisTK.jar] \
-T PrintReads \
-I <all_indvs.gatk.realigned.bam> \
-R <assembled_genome.fasta> \
-BQSR <all_indvs.gatk.bqsr.grp> \
-o <all_indvs.gatk.realigned.bqsr.bam>
```

Genotyping

All 70 RILs and two parents were genotyped at all variant sites in the final call set, using the re-aligned and base-quality recalibrated BAM file as input to GATK Unified Genotyper.

Input:
- all_indvs.gatk.realigned.bqsr.bam
- indvs.gatk.final.pass.rmdup.vcf
- assembled_genome.fasta

Output:
- all_indvs.gatk.ug.gtyp.vcf

```
java [GenomeAnalysisTK.jar] \
-T UnifiedGenotyper\
-I  <all_indvs.gatk.realigned.bqsr.bam> \
-R <assembled_genome.fasta> \
--genotyping_mode GENOTYP_GIVEN_ALLELES \
--alleles <indvs.gatk.final.rmdup.vcf>
-glm BOTH \
-nt 4  -dcov 50 \
-stand_emit_conf 4 -stand_call_conf 4 \
-o <all_indvs.gatk.ug.gtyp.vcf>
```

To ensure genotyping quality, we independently genotyped all the data using SAMtools. The SAMtools-generated genotypes were used later as one of the inputs to our identification of split loci.

Genotyping

Input:
all_indvs.gatk.realigned.bqsr.bam
assembled_genome.fasta
Intermediate:
all_indvs.smt.gtyp.final.bcf
Output:
all_indvs.smt.gtyp.final.vcf

```
[samtools] mpileup \
<assembled_genome.fasta> \
<all_indvs.gatk.realigned.bqsr.bam> | \
[bcftools] view -bvcg - \
<all_indvs.smt.gtyp.final.bcf>

[bcftools] view <all_indvs.smt.gtyp.final.bcf> \
<all_indvs.smt.gtyp.final.vcf>
```

After Genotyping

We noticed that some variant sites had three alleles identified, with only two of them showing up in the genotypes. To make things simpler, we fixed such sites by removing the third allele.

Input:
all_indvs.gatk.ug.gtyp.vcf
Output:
all_indvs.gatk.ug.gtyp.fixed.vcf
targets.report

```
python [fix_multiallelic_sites.py] \
-raw_vcf <all_indvs.gatk.ug.gtyp.vcf> \
-fixed_vcf <all_indvs.gatk.ug.gtype.fixed.vcf> \
-targets <targets.report>
```

After Genotyping

Within the genotyped VCF file, we have lost the information about sites having passed or failed VQSR. We thus annotated its FILTER fields with “PASS” or “FILTER”, based on our final call-set obtained previously.

Input:
all_indvs.gatk.ug.gtyp.fixed.vcf
indvs.gatk.final.vcf
Output:
all_indvs.gatk.ug.gtype.fixed.ann.vcf

```
python [annotate_FILTER_field.py] \
-raw_vcf <all_indvs.gatk.ug.gotyp.fixed.vcf> \
-vqsr_vcf <indvs.gatk.final.vcf> \
-out <all_indvs.gatk.ug.gtype.fixed.ann.vcf>
```

Split Loci Identification

We identified candidate alleles that had been erroneously split into separate loci by first aligning our assembled genome against itself using LASTZ (v1.02.00). We specified CIGAR as the output format, and considered only alignments with at least 90% identity. Note that "[multiple]" in the command-line setting below is part of the syntax required by LASTZ, rather than our convention stated at the very beginning of this manual.

Input:
assembled_genome.fasta

Output:
assembly_self_90.cigar.aln

```
[lastz]
<assembled_genome.fasta>[multiple] \
<assembled_genome.fasta> \
--notrivial --strand=both --chain \
--format=cigar --ambiguous=n \
--identity=90 > <assembly_self_90.cigar.aln>
```

Our home-brew python scrpt, split_loci_identifier.py, then combined the alignment, the genotypes, and the BAM file of all individuals to report a list of candidates. Command-line settings for using the script are shown below.

Split Loci Identification

Input:

assembled_genome.fasta
assembly_self_90.cigar.aln
all_indvs.gatk.ug.gtyp.fixed.ann.vcf
all_indvs.smt.gtyp.final.vcf
all_indvs.gatk.realigned.bqsr.bam

Output:

split_loci.report
proposed.genotypes

```
python [split_loci_identifier.py] \
-assembly <assembled_genome.fasta> \
-aln <assembly_self_90.cigar.aln> \
-gatk_vcf <all_indvs.gatk.ug.gtyp.fixed.ann.vcf> \
-smt_vcf <all_indvs.gatk.smt.final.vcf> \
-bam_file <all_indvs.gatk.realigned.bqsr.bam> \
-bam_dir <splitted_bam> \
-out_dir <outdir>
> <split_loci.report>
```

Our script works by first identifying variant sites where no more than 5% individuals are genotyped as heterozygous. There were individuals whose genotypes obtained from GATK UnifiedGenotyper were not consistent with ones obtained from SAMtools--we handled such cases by marking the inconsistent genotypes as missing data.

Next, our script tries to identiy pairs of duplicated loci that have (i) an alignment of at least 1000 bp, (ii) a minimum of 90% alignment identity between them, and (III) a total combined coverage at least 600X (median coverage of a locus is 350X). It then proceeds to locate variants within those pairs of homologous loci. For each variant found in one locus, its relative position within the other locus is mapped. Pileups are then generated for each paired variant sites. If one allele is erroneously split into two copies, the coverage at each is expected to be half of the average depth. With the [continued on next page]

Split Loci Identification

current version of our program, we consider a pair of variants conforming to our coverage expectation if and only if the sum of coverage at homologous positions is no more than 7X. Such a threshold will be customizable through the command-line in the future.

Our program further asks whether those pairs of variants segregate as indels, with the insertion state in one copy associated with the deletion state in the other copy within a single individual. The number of pairs of variants following both coverage and genotype patterns is then calculated for each pair of duplicated loci. Our program reports a pair of duplicated loci to be erroneously split if and only if all pairs of variants within them conform to the expected coverage and genotype patterns.

Under the output directory, specified by the "-outdir" option, the program outputs two subfolders with pileup files for duplicated loci and pairs of variants at homologous positions, respectively. "-bam_dir" asks for a path to a directory storing all the BAM files by scaffolds.

Collapsed Locus Identification

We identified paralogous loci that had been erroneously collapsed into a single locus during assembly using our home-made python script. The script takes the genotypes and the BAM file of all individuals as input to report a list of candidates. Command-line settings for using the script is shown below.

Input:

assembled_genome.fasta
all_indvs.gatk.ug.gtyp.fixed.ann.vcf
all_indvs.gatk.realigned.bqsr.bam

Output:

collapsed_loci.report

```
python [merged_loci_identifier.py] \
-bam <all_indvs.gatk.realigned.bqsr.bam> \
-assembly <assembled_genome.fasta> \
-gt <all_indvs.gatk.ug.gtyp.fixed.ann.vcf> \
-min_len 1000 \
-min_cov 650 \
-max_cov 1100 \
-het 90 \
-outdir <outdir>
```

Our program works by first identifying variant sites where (i) at least 90% of individuals are heterozygous and (ii) no more than 25% of individuals are missing their genotypes. It then proceeds to consider regions in our assembled genome (i) that contain the variant sites identified above, (ii) whose coverages range from 650X to 1100X, and (iii) whose length is greater than 1000 bp.

All these rules are customizable through the command-line as shown on the right.

Before the Linkage Map

Before building a genetic map, we first converted genotypes in the VCF file into a table. Markers with identical genotypes across all 70 RILs were collapsed into a single marker in order to reduce redundant genotype information from closely linked sites.

To minimize mapping errors we only used variant sites (i) that passed VQSR, (ii) with no more than 20% of individuals missing their genotypes, and (iii) where at least 95% of individuals are homozygous.

Further, we did not use the genotypes at markers that were located within the split loci identified above. Instead we updated them by merging their genotypes with ones at their homologous positions. The "proposed.genotypes" file is part of the output from identifying split loci.

The whole process was accomplished using the following python script below.

Input:

all_indvs.gatk.ug.gtyp.fixed.ann.vcf
proposed.genotypes

Output:

all_indvs.mstmap.gtable

```
python [vcf_parser.py] \
-analysis prepare_mapping \
-vcf <all_indvs.gatk.ug.gtyp.fixed.ann.vcf> \
-p <all_indvs> \
-gt_proposal <proposed.genotypes> \
-gt_outfmt mstmap
```

Building the Linkage Map

We then used the genotype table as input to MSTMap. The parameters we used are as follows:

population_type RIL8
population_name c_elegan
distance_function kosambi
cut_off_p_value 0.00000001
no_map_dist 10.0
no_map_size 2
missing_threshold 0.15
estimation_before_clustering yes
detect_bad_data yes
objective_function COUNT
number_of_loci 2658
number_of_individual 70

Note that we ran MSTMap with different settings until we got six major linkage groups.

Input:
all_indvs.mstmap.gtable
Output:
all_indvs.mstmap.map

```
[MSTMap.exe] \
<all_indvs.mstmap.gtable>  \
<all_indvs.mstmap.map>
```

Before Phasing

We then parsed our linkage map to get the order and orientation of scaffolds within each of the linkage groups. The python used for this purpose is not available at this stage. But the resulting file, “predicted.lgs” is found within our script package.

Before determine the haplotypic phase of our variant sites, we prepared the genotype data into another format required by the phasing software BEAGLE. To minimize phasing error we only used variant sites (i) where no more than 5% individuals are heterozygous, and (ii) at least 95% individuals had their genotype available.

Command-line settings for using our python script shows below.

Input:

- all_indvs.gatk.ug.gtyp.fixed.ann.vcf
- predicted.lgs

Output:

- lg_0.bgl.unphased
- lg_1.bgl.unphased
- lg_2.bgl.unphased
- lg_3.bgl.unphased
- lg_4.bgl.unphased
- lg_5.bgl.unphased

```
python [vcf_parser.py] \
-analysis prepare_phasing \
-vcf <all_indvs.gatk.ug.gtyp.fixed.ann.vcf> \
-p <lg> \
-known_LGs <predicted.lgs> \
-phasing_outfmt beagle
```

Phasing

Phasing was done separately for each linkage group. The command-line for one of them is shown below.

Input:

lg_0.bgl.unphased

Output:

lg_0.bgl.phased

other results from BEAGLE

```
java -jar [beagle.jar] \
unphased=<lg_0.bgl.unphased> \
missing=? \
out=<lg_0.bgl> \
niterations=30 \
nsamples=70
```
